# Gene Electrotransfer of Plasmid-Encoding IL-12 Recruits the M1 Macrophages and Antigen-Presenting Cells Inducing the Eradication of Aggressive B16F10 Murine Melanoma

**DOI:** 10.1155/2017/5285890

**Published:** 2017-05-16

**Authors:** Ursa Lampreht Tratar, Luisa Loiacono, Maja Cemazar, Urska Kamensek, Vito Michele Fazio, Gregor Sersa, Emanuela Signori

**Affiliations:** ^1^Department of Experimental Oncology, Institute of Oncology Ljubljana, Zaloska 2, Ljubljana, Slovenia; ^2^Laboratory of Genetic and Clinical Pathology, University Campus Bio-Medico of Rome, Via Álvaro del Portillo 21, 00128 Rome, Italy; ^3^Laboratory of Oncology, IRCCS “Casa Sollievo della Sofferenza”, Viale dei Cappuccini, 71013 San Giovanni Rotondo, Italy; ^4^Faculty of Health Sciences, University of Primorska, Polje 42, Izola, Slovenia; ^5^National Research Council-Institute of Translational Pharmacology (CNR-IFT), Via Fosso del Cavaliere 100, Rome, Italy

## Abstract

Cancer immunotherapy is currently one of the leading approaches in cancer treatment. Gene electrotransfer of plasmids encoding interleukin 12 (IL-12) into the cells leads to the production of IL-12, which drives immune cell polarization to an antitumoral response. One of the cell types that shows great promise in targeting tumor cells under the influence of IL-12 cytokine milieu is that of macrophages. Therefore, the aim of this study was to evaluate gene electrotransfer of antibiotic resistance-free plasmid DNA-encoding murine IL-12 (mIL-12) in mice bearing aggressive B16F10 murine melanoma. IL-12 electrotransfer resulted in the complete long-term eradication of the tumors. Serum mIL-12 and murine interferon *γ* (mIFN*γ*) were increased after IL-12 gene electrotransfer. Further on, hematoxylin and eosin (HE) staining showed increased infiltration of immune cells that lasted from day 4 until day 14. Immunohistochemistry (IHC) staining of F4/80, MHCII, and CD11c showed higher positive staining in the IL-12 gene electrotransfer group than in the control groups. Immune cell infiltration into the tumors and the high density of MHCII- and CD11c-positive cells suggest an antitumor polarization of macrophages and the presence of antigen-presenting cells that contributes to the important antitumor effectiveness of IL-12.

## 1. Introduction

In the last decade, one of the main focuses of cancer research has been the involvement of immune system in the progression of the disease. It is well established that the mechanisms of immune evasion play an important role in cancer progression. Tumor cells are able to silence the immune system, reduce the expression of tumor antigens, inactivate immune cells, and induce the microenvironment to release immune suppressors [1]. Therefore, treatments aiming at modification of immune response have become a promising approach in treatment of tumors.

Immunotherapy protocols can enhance the capacity of the immune system to fight cancer and counteract suppressing signals produced by tumor cells [2, 3]. Macrophages represent one of the main targets of immunotherapy, since they can be key actors in tumor progression. Tumor-associated macrophages (TAMs) are the most abundant cells in a tumor microenvironment. Schematically, two main TAM phenotypes could be identified in the complex shadows of their functional states: M1, with antitumor activity, and M2, with protumor activity. The protumor activities of M2 TAMs are assigned to specific subpopulations of macrophages and consist of suppression of T-cell response, angiogenesis, tumor cell invasion, motility, and intravasation [4]. M1 macrophages are activated by the granulocyte monocyte colony-stimulating factor (GM-CSF) and the tumor necrosis factor alpha (TNF-*α*). They express MHCII and produce IL-12 and IL-23, reactive oxygen species (ROS), inflammatory cytokines (IL-1*β*, TNF, and IL-6), and antitumoral chemokines (CXCL-9 and CXCL-10) attracting Th1 cells to eliminate tumor cells. On the contrary, M2 macrophages are induced by IL-4, IL-13, IL-21, and IL-33, and they release IL-10 and CCL-17 and CCL-22 and CCL-24 chemokines that recruit Tregs and Th2 cells to promote tumor growth: they are weak antigen presenters, inhibiting inflammation and stimulating angiogenesis and tissue remodeling [5, 6]. Due to the central role of the immune system and macrophages in tumor promotion and progression, current studies are focused on immunotherapy strategies with the aim of testing translational preclinical protocols to propose to human patients. These kinds of treatments can take advantage of gene transfer based on plasmid DNA vectors, carrying tumor antigens or immunomodulatory molecules to stimulate the immune system [7, 8].

In particular, gene electrotransfer (GET) is an electroporation-based technique able to boost the efficacy of naked-DNA transfer by the application of pulsed electric fields (electroporation (EP)) after the injection of the plasmid vector [9]. Besides helping DNA delivery into the target cells, GET exerts evident adjuvant effects: the stress condition stimulates the local production of inflammatory cytokines that activate the innate immune reaction with the recruitment, among others, of M1 macrophages. Adaptive immunity is also stimulated with the attraction of lymphocytes and inflammatory cells to the electroporated areas [10]. Recently, the effect of IL-12 immunogene transfer has been extensively investigated as an antitumor therapy: IL-12's ability to activate innate as well as adaptive immunity has been demonstrated in different types of tumors in preclinical and clinical studies [11–13]. IL-12's main function is promoting the cytotoxicity of immune cells of both the innate and adaptive immune systems. Specifically, IL-12-induced secretion of IFN*γ* from NK cells and T lymphocytes boosts the function of antigen-presenting cells by increasing the class I and II MHC molecule expressions. In addition, IL-12 also promotes an antitumor type 1 cytokine environment [14]. Gene electrotransfer with plasmid-encoding IL-12 has been tested as an experimental tumor treatment in various studies in induced subcutaneous tumors and lung metastases in mice [15–17]. Results of these studies show a systemic and local increase of IL-12 and consequently also of the potent antitumor cytokine IFN*γ* [18]. The IL-12 GET, regardless of the site of delivery (intratumorally, intramuscularly, or peritumorally), induced significant tumor growth delay and even complete regression of the tumor, which was the highest (up to 90%), when plasmid-encoding IL-12 was injected intratumorally [15–18]. Furthermore, infiltration of T lymphocytes, natural killer cells, and dendritic cells, as well as a reduced number of blood vessels, were determined in tumors, indicating also an antiangiogenic effect mediated by IL-12. In addition, the circulating lymphocytes after the IL-12 gene electrotransfer were measured [19–21]. However, the role of macrophages in the antitumor effect of IL-12 GET has not been evaluated yet.

Plasmid used in the abovementioned studies encoded IL-12, whose expression was regulated by different strong constitutive promoters, and the ampicillin antibiotic resistance gene was incorporated in the plasmid backbone as a selection marker. However, one of the main concerns of the regulatory agencies (EMA, FDA) about the use of plasmid DNA in gene therapy clinical trials is the antibiotic resistance selection marker [22, 23]. To ease the translation into human clinical trials of preclinical protocols based on plasmids delivered by GET, there is a need to develop advanced types of plasmids. One possibility is the use of plasmids carrying genes for antibiotic resistance not used in current clinical practice, such as kanamycin. Nevertheless, the most preferable option is the use of plasmid DNA without any gene encoding an antibiotic resistance.

Therefore, the aim of this study was to evaluate the antitumor effectiveness of plasmid-encoding murine IL-12, lacking an antibiotic resistance gene, in an aggressive murine melanoma after intratumoral GET. To this end, B16F10 melanoma tumor bearing mice were intratumorally injected with IL-12 plasmid vector by electrotransfer. The tumor growth and the elicited immune response were evaluated at different posttreatment time points, with a special focus on the macrophage-mediated immune response.

## 2. Material and Methods

### 2.1. Plasmid DNA

**Figure 1:**
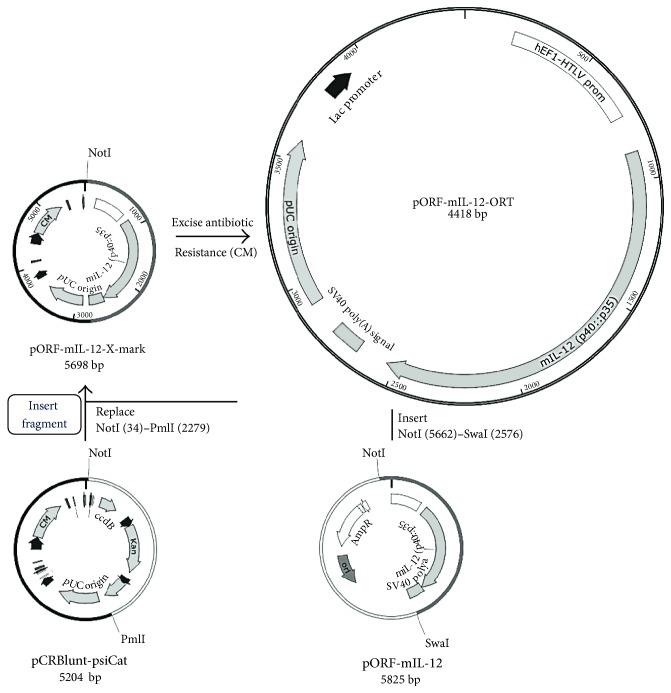
Construction of plasmid pORF-mIL-12-ORT by standard cloning methods and operator-repressor titration (ORT) technology. AmpR: ampicillin resistance gene; CM: chloramphenicol resistance gene; Kan: kanamycin resistance gene.

Two plasmids were used in this study: pORF-mIL-12-ORT, encoding a mouse IL-12 gene and lacking an antibiotic resistance gene, and pControl that served as a control plasmid without any therapeutic genes. The construction of pControl was described in our previous study [24]. To construct pORF-mIL-12-ORT, standard cloning methods and operator-repressor titration (ORT) technology [25, 26] were used, followed by transformation into competent *(E. coli*) cells (Figure 1). The source plasmid for IL-12 was a plasmid-encoding mouse IL-12 fusion gene under the control of a constitutive hybrid promoter EF-1*α*/HTLV (pORF-mIL-12, Invivogen, Toulouse, France). The pCRBluntPsiCat X-mark plasmid (Cobra Biologics, Keele, UK) was used to prepare the antibiotic-free plasmid. The mIL-12 expression cassette was cloned into the pCRBluntpsiCat plasmid using *NotI*, *SwaI*, and *PmlI* restriction enzymes, and the antibiotic resistance-free plasmid was produced using the X-mark™ technology and antibiotic-free maintenance system ORT® (Cobra Biologics). The restriction enzymes, Ligation Kit, Gel Extraction Kit, Plasmid Miniprep Kit, and TransformAid Bacterial Transformation Kit together with the *E. coli* strain *JM107* were all purchased from Thermo Fisher Scientific (Waltham, MA, USA). The X-mark and ORT Technologies were obtained from Cobra Biologics (Keele, UK). The newly constructed plasmid was confirmed by restriction analysis and sequenced using MacroGen services.

All plasmids were isolated and purified using an EndoFree Plasmid Mega Kit (Qiagen, Hilden, Germany) according to the instructions provided with the kit. The plasmid DNA was eluted in Endotoxin-free water (Qiagen) to a concentration of 1 mg/ml. The purity and concentrations were determined spectrophotometrically (Epoch Microplate Spectrophotometer, Take3™ Micro-Volume Plate, BioTek, Bad Friedrichshall, Germany). Additionally, the concentration and identity were confirmed by restriction analysis on an electrophoretic gel.

### 2.2. Mice and Tumors

The experiments performed in this study were in compliance with the guidelines for animal experiments of the EU directive (2010/63/EU) and with the permission of the Veterinary Administration of the Ministry of Agriculture, Forestry and Food of the Republic of Slovenia (Permission Number 34401-1/2015/7). The animals used in the experiments were 6–8-week old female C57Bl mice (Envigo, Udine, Italy). The mice were quarantined for a period of 2 weeks before the experiments began. The mice were maintained in a 12 h light/dark cycle under specific pathogen-free conditions at a constant room temperature and humidity. Food and water were provided ad libitum. For the induction of subcutaneous tumors, a suspension of 1 × 10^6^ B16F10 cells, prepared from cell cultures in vitro in 0.1 ml of physiological solution, was injected subcutaneously into the right flank of the mice. When the tumors reached 40 mm^3^ volume, the animals were randomly divided into experimental groups and subjected to a specific experimental protocol. When the tumors reached 350 mm^3^, the mice were euthanized. Additionally, two groups of mice without tumors were used as an internal control.

### 2.3. Electroporation Protocol

In vivo GET of plasmid DNA into B16F10 tumors was performed once, when the tumor volume reached 40 mm^3^ with an intratumoral injection of 25 *μ*l (2 *μ*g/*μ*l) of plasmid (50 *μ*g in total) in endotoxin-free water (H_2_O). Additionally, as a positive control, an injection of 25 *μ*l of lipopolysaccharide LPS (Sigma-Aldrich, Missouri, USA) at a concentration of 2 mg/ml (50 *μ*g in total) was injected into the skin of non-tumor-bearing mice. The experiments on tumor-bearing mice were performed independently twice. The experiment performed on non-tumor-bearing mice was performed once with 2 mice per negative control group and 8 mice per positive control group. The experimental groups of non-tumor-bearing mice were no injection (negative control) and intradermal injection of lipopolysaccharide (LPS) (positive control). The experimental groups of tumor-bearing mice were injection of endotoxin-free water alone (H_2_O) or in combination with the application of electric pulses (H_2_O + EP) and injection of control plasmid pControl combined with the application of electric pulses (pControl + EP group) and therapeutic plasmid pORF-mIL-12-ORT combined with the application of electric pulses (pORF-mIL-12-ORT + EP). Each experimental group was further divided into groups depending on the time of sample collection (day 1, day 4, and day 8 for all groups and an additional 11 days for the pControl + EP group and 14 days for the pORF-mIL-12-ORT + EP and LPS (positive control) groups). The absence of 14 days for all the groups except the pORF-mIL-12-ORT + EP and LPS (positive control) groups and an additional day 11 for the pControl + EP group was due to the rapid tumor growth. The mice were euthanized when the tumor mass reached the humane endpoint volume of 350 mm^3^. Altogether, the number of mice per time point for each experimental group was 4–7 except for the LPS (positive control) group where the number was 2 (Table 1). Additionally, 8 mice were included in the pORF-mIL-12-ORT + EP group to determine the effect of therapy on long-term tumor regression or recurrence. Electric pulses generated by an electroporator ELECTRO cell B10 HVLV (BETA tech, Saint-Orens-de-Gameville, France) were delivered 10 min after injection of plasmid DNA through two parallel stainless steel electrodes with a 6 mm distance between them. Eight square-wave electric pulses with an amplitude of 360 V (amplitude over distance ratio 600 V/cm), duration of 5 ms, and at a frequency of 1 Hz, given in a perpendicular direction, were applied to the tumor. To enable better contact of the electrodes with the skin overlying tumor, a conduction gel was applied to the skin.

### 2.4. Tumor Growth

The therapeutic effect of gene electrotransfer was assessed by measuring the tumor size every other day after the therapy using a Digital Vernier Caliper. Tumor volume was calculated according to the formula for an ellipsoid: *V* = *axbxc* *π*/6, where a, b, and c represent perpendicular tumor diameters [27]. From those, tumor-doubling times representing the time in which the tumors doubled the volume at the beginning of the therapy were calculated. Tumor growth curves were drawn as arithmetic means (AM) with bars representing standard errors (SEM). The weight of the mice was followed as a general index of systemic toxicity.

### 2.5. ELISA Assay

Blood was collected from the same animals that were used for histological analysis from the infraorbital sinus into serum collection tubes. After that, the blood was allowed to coagulate for 2 h and centrifuged for 5 minutes at 2000 rfc in order to obtain the serum. For determination of mIL-12 and mIFN*γ* in mice, sera ELISA (Mouse IL-12 Quantikine ELISA Kit and Mouse IFN*γ* Quantikine ELISA Kit, both R&D Systems, Minneapolis, MN, USA) was performed according to the manufacturers' instructions.

### 2.6. Histology and Immunohistochemistry

At different time points after treatment (1, 4, 8, 11, and 14 days) 2–7 mice from each experimental group were sacrificed and the tumors excised. The tumors were fixed in IHC zinc fixative (BD Biosciences, New Jersey, USA) and embedded in paraffin. Five consecutive 2 *μ*m thick sections were cut from each paraffin block. The first section was stained using hematoxylin (H) and eosin (E) according to standard histochemical procedures. The following sections were used for IHC staining with primary antibodies to identify macrophages (F4/80), major histocompatibility complex II (MHCII), and dendritic cells (CD11c) or without a primary antibody as a negative control.

Immunohistochemistry was performed on the tissue sections after antigen retrieval in a sodium citrate buffer (10 mM sodium citrate, pH 6), for 60 minutes at 95°C for CD11c antibody, and without antigen retrieval for F4/80 and MHCII antibodies. The samples were incubated with Rat anti-Mouse F4/80 (BM8, eBioscience, San Diego, USA, 1 : 50); Rat anti-Mouse MHCII (M5/114.15.2, eBioscience, 1 : 100); and Armenian Hamster anti-Mouse CD11c (N418, AbCam, Cambridge, United Kingdom, 1 : 100) antibodies overnight at 4°C. Then, the sections were incubated for 30 minutes with biotin-labelled secondary antibodies anti-Rat IgG (Santa Cruz, California, USA, 1 : 1000) and anti-Hamster IgG (eBioscience, San Diego, USA, 1 : 1000) and with HRP-conjugated avidin for 30 minutes at room temperature (ABC Staining Kit sc-2019, Santa Cruz). Detection was achieved using a substrate/chromogen mixture (DAB) and hematoxylin counterstaining. Incubation with the primary antibody was omitted for the negative controls. The IHC-stained slides were observed under light microscopy, and at least 3 images of viable tumor tissue from each slide were captured with a DP72 CCD camera (Olympus, Tokyo, Japan) connected to a BX-51 microscope (Olympus) under 20x magnification (numerical aperture of 0.85). IHC grading was performed by 3 independent investigators based on the estimation of intensity and number of positive cells. A semiquantitative scoring system for immunopositive cells ranging (+) for low (less than 30%), (++) for moderate (31–60%), and (+++) for high (more than 61%) positivity as described previously [28, 29].

### 2.7. Statistical Analysis

All data were tested for normality of distribution with the Shapiro-Wilk test. The differences between the experimental groups were statistically evaluated by one-way analysis of variance (one-way ANOVA) followed by a Holm-Sidak test for multiple comparisons. A *P* value of less than 0.05 was considered to be statistically significant. SigmaPlot Software (Systat Software, San Jose, CA, USA) was used for graphical representation and statistical analysis.

## 3. Results

### 3.1. Complete Murine Melanoma Regression after IL-12 GET

**Figure 2:**
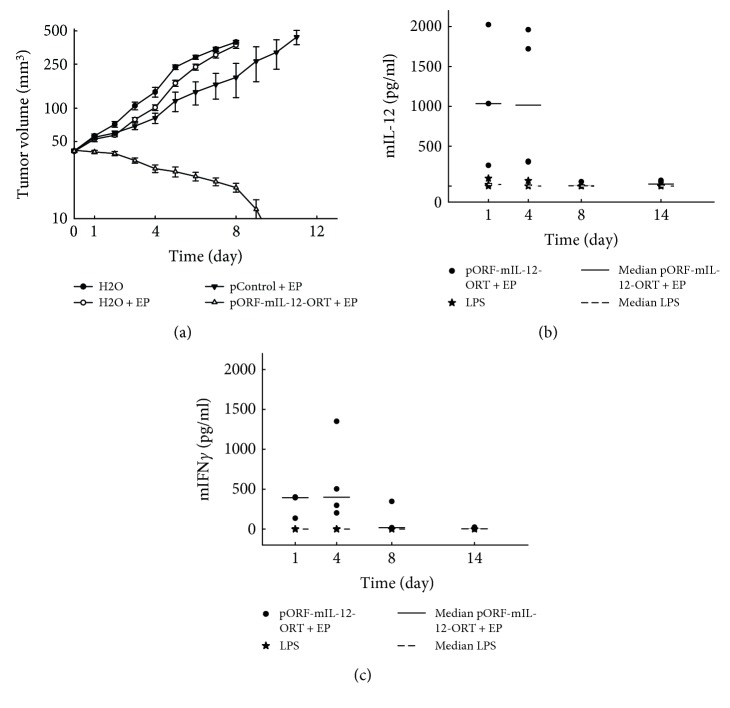
(a) Tumor growth of B16F10 tumors in C57/Bl6 after IL-12 GET compared to that in the control animal groups. The experiments were performed independently twice. (b) ELISA assay for the detection of mIL-12 in the LPS group (positive control) on days 1, 4, and 8 and in the pORF-mIL-12-ORT + EP group on days 1, 4, 8, and 14. (c) Detection of mIFN*γ* in the LPS and pORF-mIL-12-ORT + EP groups on days 1, 4, 8, and 14 using an ELISA assay.

In this study, aggressive malignant melanoma tumors B16F10 were used to evaluate the efficacy of GET with a plasmid-encoding mouse IL-12 without the antibiotic resistance gene. When tumors reached 40 mm^3^, the therapy was performed and from that moment on, the tumors' diameters were measured every other day. Tumor-doubling time in the control group (H_2_O) was 2.0 ± 0.4 days, while in the groups treated with EP (H_2_O + EP), it was 2.7 ± 0.5 days. By day 8, all the mice from the H_2_O and H_2_O + EP groups reached the size of humane endpoint volume of 350 mm^3^ and were euthanized. The tumor-doubling time in the control plasmid group was 3.5 ± 1.0 days. By day 11, all the tumors from this group reached the endpoint volume and the animals were euthanized. Tumors treated with gene electrotransfer of pORF-mIL-12-ORT completely responded to the therapy and by day 14 were no longer palpable (Figure 2(a)). Treated animals were followed regularly for the possible recurrence of the tumors. Importantly, a complete response was present throughout the observation period (85 days).

### 3.2. Intratumoral IL-12 GET Increases Circulating mIL-12 and mIFN*γ* Levels

The expected consequence of gene electrotransfer of plasmid-encoding mIL-12 is the increase of mIL-12 level and consequently of mIFN*γ*. Therefore, to evaluate whether intratumoral GET electrotransfer of mIL-12 also leads to the systemic release of cytokines, ELISA tests for mIL-12 and mIFN*γ* were performed. The serum levels of mIL-12 and mIFN*γ* were measured at different time points after the therapy. In the case of mIL-12, we observed an increase in the amount of mIL-12 in the therapeutic group of pORF-mIL-12-ORT + EP on day 1 and day 4, where the average amount of mIL-12 on day 1 was 1105 pg/ml ± 884 pg/ml and on day 4 1073 pg/ml ± 891 pg/ml. On the same days, we registered an increase in the group treated with LPS (positive control group), even though with lower levels with respect to the therapeutic group. The average amount of mIL-12 was 39 pg/ml ± 51 pg/ml on day 1 and 23 pg/ml ± 39 pg/ml (Figure 2(b)) on day 4. Due to the high variety of data in the therapeutic group, the difference in average values of the therapeutic and LPS groups did not reach statistical significance. In the case of mIFN*γ*, the levels measured after LPS treatments were below the limit of detection. However, measurable mIFN*γ* levels were determined in the therapeutic group pORF-mIL-12-ORT EP. The mIFN*γ* was measurable on days 1, 4, and 8, and the peak of mIFN*γ* was detected on day 4 with an average amount of 587 pg/ml ± 523 pg/ml (Figure 2(c)).

### 3.3. IL-12 GET Induces an Enhanced Intratumoral Infiltration of M1 Macrophages and Antigen-Presenting Cells

In order to evaluate the immune response and determine infiltration of immune cells, HE and IHC staining were performed: by means of HE staining, the amount of immune cell infiltration in the tumor and surrounding tissues in different groups was observed; by IHC staining with F4/80, MHCII and CD11c, macrophages and dendritic cells were detected for the immune cell infiltrates evaluation as reported elsewhere [30]. Specifically, dendritic cells were detected by CD11c staining, and macrophages were detected by IHC staining with F4/80 as a generic marker and MHCII-based IHC was used to distinguish between M1 (high expressing) and M2 (low expressing) macrophages [31]. Tumor and skin samples were planned to be excised in all experimental groups on days 1, 4, 8, and 14. Due to the rapid tumor growth in the H_2_O, H_2_O + EP groups, the mice were euthanized on day 8 when the tumors reached their endpoint size, so it was not possible to collect the tumors on day 14 posttreatment. In the case of the pControl + EP group, tumors were collected on day 11 instead of day 14 for the same reason. The pORF-mIL-12-ORT + EP group was excised on day 14.

**Figure 3:**
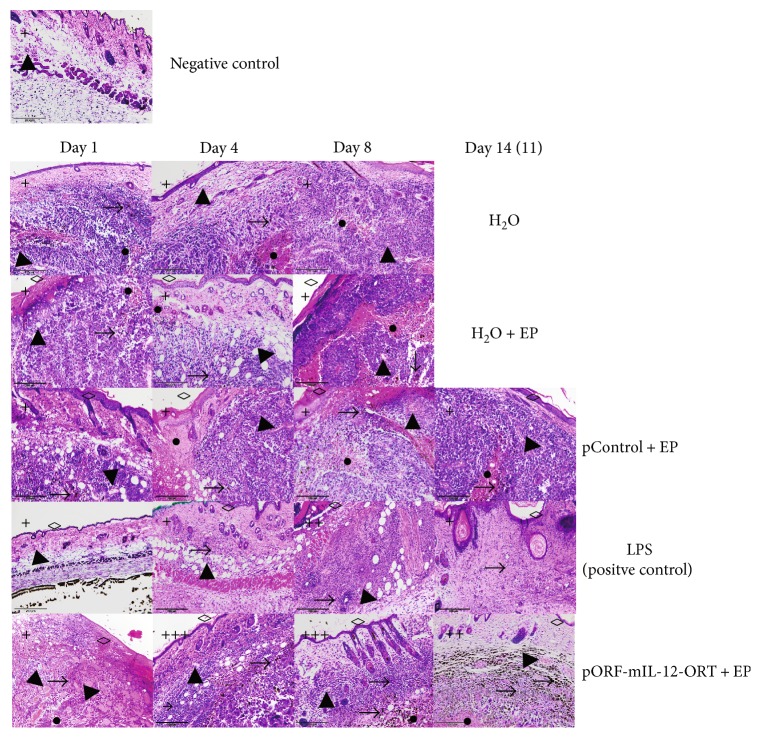
Representative images of hematoxylin/eosin staining of all the experimental groups (negative control (only skin) with no specific day and H_2_O, H_2_O + EP, pControl + EP, LPS (positive control), and pORF-mIL-12-ORT + EP) on day 1, day 4, day 8, and day 14 (11). A semiquantitative scoring system for immunopositive cells was used: (+) low, (++) moderate, and (+++) high positivity. Various histological characteristics of the figures are marked: immune cell infiltration (**→**), blood vessels (▲), melanin (**→**), necrosis (●), and epithelium damage (◊). The images were taken under 20x magnification (numerical aperture 0.85).

In the LPS (positive control) group, HE staining revealed an infiltration of immune cells in the region of the dermis that was very scarce on day 1, reached its peak on day 4, and lasted up until day 14 with the highest density of immune cells on day 4 and day 8. The immune cells that infiltrated into the pORF-mIL-12-ORT + EP group were more concentrated in the tumor region, following the same trend of intensity of the LPS group, but in a more pronounced way. In the other groups, little or no infiltration of the immune cells was observed (Figure 3).

**Figure 4:**
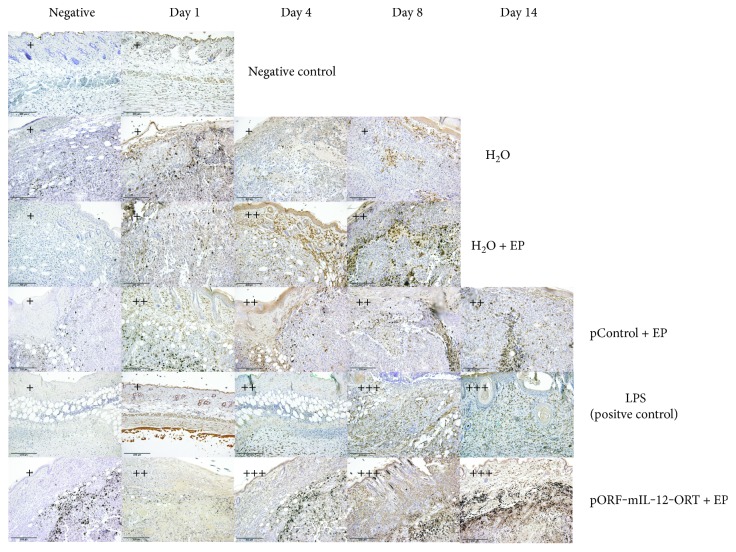
Immunohistochemical staining of F4/80-positive cells of all the experimental groups (negative control, H_2_O, H_2_O + EP, LPS (positive control), pControl + EP, and pORF-mIL-12-ORT + EP) on day 1, day 4, day 8, and day 14 (11). A semiquantitative scoring system for immunopositive cells was used: (+) low, (++) moderate, and (+++) high positivity. The negative images of all groups are not related to a specific day. The images were taken under 20x magnification (numerical aperture 0.85).

IHC for F4/80 showed a positive staining in the groups of H_2_O + EP, pControl + EP, pORF-mIL-12-ORT + EP, and LPS (positive control), whereas no signal was observed in the groups of H_2_O and negative control—skin. The signal appeared more intense in the group of LPS and pORF-mIL-12-ORT + EP compared to that in the H_2_O + EP and pControl + EP, with a peak on days 4, 8, and 14 (Figure 4).

**Figure 5:**
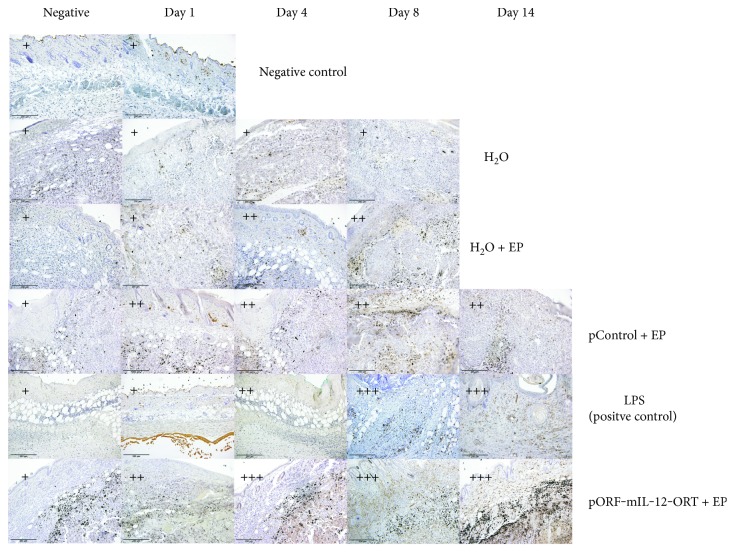
Immunohistochemical staining of MHCII-positive cells of all the experimental groups (negative control, H_2_O, H_2_O + EP, LPS (positive control), pControl + EP, and pORF-mIL-12-ORT + EP) on day 1, day 4, day 8, and day 14 (11). A semiquantitative scoring system for immunopositive cells was used: (+) low, (++) moderate, and (+++) high positivity. The negative images of all groups are not related to a specific day. The images were taken under 20x magnification (numerical aperture 0.85).

IHC for MHCII again showed a positive staining in the H_2_O + EP, pControl + EP, pORF-mIL-12-ORT + EP, and LPS (positive control) groups, whereas in the H_2_O and negative control—skin groups with no positive staining were registered. The intensity of MHCII-positive staining was stronger in the pORF-mIL-12-ORT + EP group and weakened from the LPS (positive control) toward the pControl + EP and H_2_O + EP groups (Figure 5). Concerning time-dependent intensity changes, the MHC-positive signal appeared more vivid on day 4 and lasted up until day 14. The pronounced positive staining of F4/80 and MHCII in the groups of pORF-mIL-12-ORT + EP and LPS indicates a higher density of M1 macrophages in these two groups.

**Figure 6:**
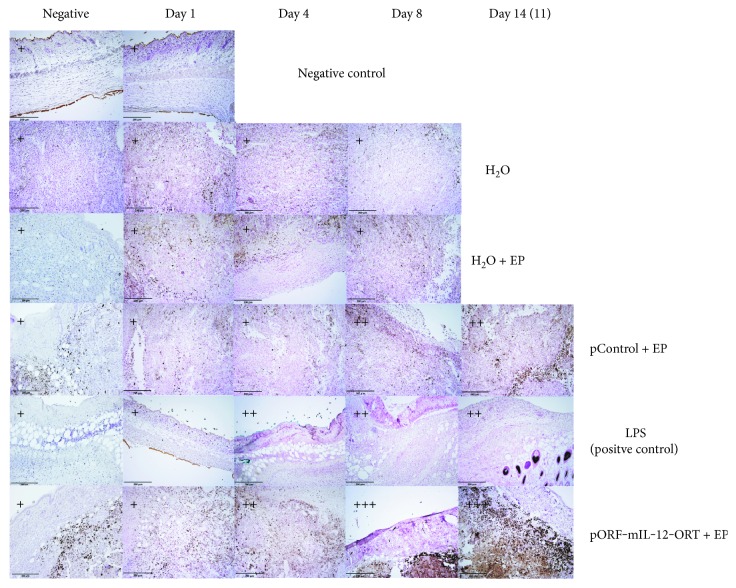
Immunohistochemical staining of CD11c-positive cells of all the experimental groups (negative control, H_2_O, H_2_O + EP, LPS (positive control), pControl + EP, and pORF-mIL-12-ORT + EP) on day 1, day 4, day 8, and day 14 (11). A semiquantitative scoring system for immunopositive cells was used: (+) low, (++) moderate, and (+++) high positivity. The negative images of all groups are not related to a specific day. The images were taken under 20x magnification (numerical aperture 0.85).

IHC for CD11c demonstrated strong positivity for the pORF-mIL-12-ORT + EP group, in which the staining intensity increased progressively from day 1 to day 14, and a positive signal for the pControl + EP and LPS groups, while the signal appeared very weak or absent in the H_2_O, H_2_O + EP, and negative groups (Figure 6). These data indicate a small accumulation of CD11c + dendritic cells in tumors treated with LPS or electroporated with the pControl plasmid and a strong infiltration of these cells in tumors treated with pORF-mIL-12-ORT in combination with electric pulses.

## 4. Discussion

The results of our study demonstrated that the use of intratumor IL-12 GET in aggressive murine melanoma tumors resulted in the complete regression of the tumors with no recurrence and high infiltration of immune cells into the tumor tissue. Additionally, an increase of serum mIL-12 and mIFN*γ* was observed which accordingly led to the polarization of macrophages into the antitumoral M1 phenotype, demonstrated by the intense MHCII staining and the accumulation of CD11c+ antigen-presenting cells in the tumor tissue. Furthermore, the constructed plasmid devoid of an antibiotic resistance gene proved its effectiveness and its applicability for further clinical studies.

The immune system plays a pivotal role in tumor elimination, and in particular, the immune cells of the innate immune system represent the first line of host immune defense. Antigen-presenting cells serve as a bridge between the innate and the adaptive systems, with their specific ability to display a foreign antigen through MHC complex to T cells which then induce the adaptive immune response. Dendritic cells and macrophages are the most well-known antigen-presenting cells among the innate immune cells. Dendritic cells in particular are of great importance since they can activate not only memory T cells, as macrophages do, but also naive T cells, with a broader range of antigen presentation. Macrophages are known to be present in normal tissues as well as in tumors in different forms: antitumoral macrophages or M1 and protumoral macrophages or M2. One of the most important differences between these two subtypes is the ability of antigen presentation which can only be exerted by M1 cells. Also, M1 macrophages induce the Th1 cells which in turn produce type 1 cytokines, known to function as antitumor molecules. Studies have already shown that intramuscular administration of EP pulses alone causes the infiltration of different immune cells such as macrophages, dendritic cells, and T cells into the muscle [16, 30]. IL-12 GET additionally increases the infiltration of immune cells into fibrosarcoma tumors [32] and specifically of CD4+ and CD8+ T cells into murine melanoma tumors [33].

In the present study, immune response was measured by the increase of two important antitumor cytokines: mIL-12 and mIFN*γ*. We showed that GET with the therapeutic plasmid increased the serum concentration of mIL-12 and mIFN*γ* and induced the infiltration of immune cells. In our study, the peak of mIL-12 was observed 1 day after GET, and the peak of mIFN*γ* 2-3 days after the peak of mIL-12. This is consistent with data in the literature [15, 16, 34] where a peak of serum mIL-12 was observed one day after gene therapy by intratumoral application of adenovirus expressing mIL-12, followed approximately two days later by a similar peak of serum mIFN*γ*. The decline of the serum concentration on day 14 correlates with the complete regression of tumors, confirming that transfected tumor cells that were producing IL-12 were indeed killed.

By HE staining, we demonstrated that infiltrating inflammatory cells were almost absent in the H_2_O and negative control—skin groups, scarce in pCONTROL + EP and H_2_O + EP treated tissues, and abundant in the dermis zone of the LPS-treated tissues (positive controls) and in the tumor region of the IL-12 GET-treated tumors, with a progressive accumulation from day 4 to day 14 and a peak on day 8. The results of the histological analysis of tissue sections indicate that infiltration of inflammatory cells into the tumor microenvironment was induced by IL-12 GET.

We investigated the identity of the immune infiltrate, revealing the presence of antigen-presenting cells by immunohistochemistry on paraffin embedded sections. A major component of the cellular infiltrate was represented by macrophages: F4/80-positive staining was seen in all four time frames in all groups apart from the H_2_O group and negative control group—skin, with an important high intensity of staining in the LPS and pORF-mIL-12-ORT + EP groups, especially on days 8 and 14. MHCII staining corresponded closely to F4/80 staining, with a higher intensity in the pORF-mIL-12-ORT + EP and LPS groups particularly on days 8 and 14. This signal overlapping indicates the recruitment of M1 macrophages from day 4 until day 14 in IL-12 GET-treated tumors and in the positive control represented by LPS-treated tissues. In contrast, the number of M1 macrophages was reduced in the pControl + EP and H_2_O + EP groups and absent in the H_2_O and negative control—skin groups. As shown by the CD11c staining, in the pORF-mIL-12-ORT + EP group, dendritic cells were massively mobilized from days 4–8 until day 14, while in the LPS and pControl + EP groups, the dendritic cell infiltrate was moderate and in the H_2_O and H_2_O + EP groups, almost absent. These data suggest a combined role for EP and IL-12 plasmid administration in recruiting immune cells, among which the antitumoral M1 macrophages and dendritic cells were detected. In the presence of the therapeutic protein, IL-12, this massive antigen-presenting cell migration at the site of injection favors tumor-associated antigen presentation to T cells.

Besides the different intensity of staining between the groups, also the onset of staining within different time frames was observed. F4/80-positive staining is shown in all four time frames in all groups, apart from the H_2_O group and negative control group—skin. Although positive staining was present, we could observe that the intensity of the staining was more intense in the LPS and pORF-mIL-12-ORT + EP groups, especially on days 8 and 14. MHCII staining showed that the presence of M1 macrophages was higher in the groups of LPS and pORF-mIL-12-ORT + EP whereas the population of M1 macrophages reduced from the LPS toward the pControl + EP and H_2_O + EP groups. Again, there was a difference between the time frames. We observed that the population of M1 macrophages was abundant on day 4 and lasted until day 14.

In conclusion, in this study, besides reinforcing the data of previous researchers' results showing an increase of immune cell infiltration after IL-12 GET [19], we focused our attention on the involvement of macrophages and dendritic cells in tumor eradication. We demonstrated a massive infiltration of immune cells, with the significant involvement of M1 macrophages and dendritic cells in tumor elimination. It is important to note that the tumors analyzed in this study are weakly immunogenic [35]; that means that by themselves, they have a low ability to induce an immune response. Since the recruitment and activation of specific types of immune cells represent a key tool for tumor elimination, with this study, we showed that by gene electrotransfer of a plasmid-encoding IL-12, we can transform a weakly immunogenic tumor into an immunogenic tumor able to induce a systemic immune response. Because a very efficient plasmid vector with no antibiotic resistance gene was employed, we are confident that our results can open a new perspective for melanoma patient treatment, where current immunotherapeutical approaches proved only modestly beneficial [36].

## Figures and Tables

**Table 1:** The experimental groups and the number of mice sacrificed at each time point.

Group		Time intervals					
		1 day	4 days	8 days	11 days	14 days	85 days
		Number of mice					
(1) Negative control group	Skin	2					
(2) Positive control group	Skin + LPS	2	2	2	0	2	0
(3) Control group: H_2_O	Tumors + endotoxin-free water application	6	6	5	0	0	0
(4) Control group: H_2_O + EP	Tumors + endotoxin-free water application + EP pulses	6	6	6	0	0	0
(5) Control group: pControl + EP	Tumors + control plasmid + EP pulses	6	6	4	5	0	0
(5) Therapeutic group: pORF-mIL- 12-ORT + EP	Tumors + therapeutic plasmid + EP pulses	6	7	6	0	7	8

## Abbreviations

AM: Arithmetic means
GET: Gene electrotransfer
GM-CSF: Granulocyte monocyte colony-stimulating factor
HE: Hematoxylin and eosin
IFN*γ*: Interferon *γ*
IHC: Immunohistochemistry
IL-12: Interleukin 12
LPS: Lipopolysaccharide
ORT: Operator repressor titration
ROS: Reactive oxygen species
SEM: Standard errors
TAM: Tumor-associated macrophages
TNF-*α*: Tumor necrosis factor alpha.
